# Intervention for Justice-Involved Homeless Veterans With Co-Occurring Substance Use and Mental Health Disorders: Protocol for a Randomized Controlled Hybrid Effectiveness-Implementation Trial

**DOI:** 10.2196/70750

**Published:** 2025-07-18

**Authors:** Kathryn Bruzios, Paige M Shaffer, Daniel M Blonigen, Michael A Cucciare, Michael Andre, Thomas Byrne, Jennifer Smith, David Smelson

**Affiliations:** 1 Center for Healthcare Organization and Implementation Research (CHOIR) Edith Nourse Rogers Memorial Veterans Hospital Bedford, MA United States; 2 University of Massachusetts Chan Medical School Worcester, MA United States; 3 Center for Innovation to Implementation Veterans Affairs Palo Alto Health Care System Palo Alto, CA United States; 4 Department of Psychiatry and Behavioral Sciences School of Medicine Stanford University Stanford, CA United States; 5 Center for Mental Healthcare and Outcomes Research Central Arkansas Veterans Healthcare System Little Rock, AR United States; 6 Veterans Affairs South Central Mental Illness Research, Education and Clinical Center Central Arkansas Veterans Healthcare System Little Rock, AR United States; 7 Department of Psychiatry University of Arkansas for Medical Sciences Little Rock, AR United States

**Keywords:** criminal justice, mental health, substance use, co-occurring disorder, veterans, hybrid trial

## Abstract

**Background:**

The US Veterans Affairs mental health residential rehabilitation treatment programs (MH RRTPs) provide residential care for veterans experiencing homelessness. However, those with co-occurring mental health and substance use disorders and criminal legal involvement require additional interventions to address risk factors for recidivism.

**Objective:**

We aimed to (1.1) evaluate whether the Maintaining Independence and Sobriety through Systems Integration, Outreach, and Networking Criminal Justice version (MISSION-CJ) intervention lowers criminal recidivism and improves health-related outcomes; (1.2) examine the mechanisms that impact outcomes; and (2) qualitatively assess the implementation of MISSION-CJ.

**Methods:**

Veterans participating in an MH RRTP (N=226) will be randomized to the enhanced usual care (EUC) or MISSION-CJ conditions in a hybrid type 1 randomized controlled trial to test the effectiveness and implementation of MISSION-CJ, a multicomponent intervention for co-occurring disorder. Both conditions will receive 6 months of services beginning within a week of MH RRTP enrollment (duration of stay: 3 months) and continue for 3 months after the MH RRTP in the community. The veterans in the EUC group (113/226, 50%) will receive a peer support curriculum and community outreach and linkage delivered by a peer support specialist. The veterans in the MISSION-CJ group (113/226, 50%) will receive team-based (case manager and peer support specialist) care, including treatment planning, case management using a critical time intervention model to promote referrals and linkages, enhanced dual recovery therapy sessions, and peer support sessions. Assessments, including questions regarding substance use and mental health history, criminal history and recidivism risk, housing, employment, medication adherence, mutual-help group attendance, antisocial attitudes, affiliations with peers, community involvement, and treatment services received, will be conducted at baseline and 6 months and 15 months after baseline. We will use generalized linear mixed effects regression models to evaluate MISSION-CJ based on outcomes (objective 1.1). We will conduct mediation analysis to examine mechanisms of action (objective 1.2). For the qualitative evaluation (objective 2), we will use thematic analysis to identify themes.

**Results:**

As of March 2025, 118 veterans (site 1: n=52, 44.1% and site 2: n=66, 55.9%) have been enrolled. Overall, 58 veterans (site 1: n=27, 47% and site 2: n=31, 53%) have been randomized to the MISSION-CJ group, and 60 veterans (site 1: n=25, 42% and site 2: n=35, 58%) have been randomized to the EUC group. Overall, 23 interviews for the qualitative evaluation have been completed with veterans. Veterans are continuing to receive treatment and completing follow-up assessments. The findings from this trial and qualitative evaluation will be available by 2026. The quantitative and qualitative components of this project are intended to work synergistically to reinforce knowledge of MISSION-CJ’s effectiveness, implementation, and scalability.

**Conclusions:**

If effective, the implementation of MISSION-CJ alongside the MH RRTPs may be advantageous to address risk factors related to recidivism.

**Trial Registration:**

ClinicalTrials.gov NCT04523337; https://clinicaltrials.gov/study/NCT04523337

**International Registered Report Identifier (IRRID):**

DERR1-10.2196/70750

## Introduction

### Background

Each year, 146,000 veterans in the United States are released from correctional settings [1], with estimates being even higher when accounting for veterans who have been diverted from incarceration. Veterans with current or previous involvement in the criminal legal system [2,3] (vs those without such involvement) have higher rates of behavioral health conditions [4], with 60% of them diagnosed with a co-occurring disorder (COD) and mental health and substance use disorder [5]. In addition, veterans with criminal legal involvement and a COD often have other needs associated with behavioral health, such as unemployment and homelessness [6], and consequently, they are at greater risk for criminal recidivism [7-9]. Veterans who are incarcerated are ineligible for Veterans Affairs (VA) services [10], rendering recidivism a critical issue to address, as it disrupts access to VA services that address substance use, mental illness, and other medical comorbidities, all of which are highly prevalent among veterans with criminal legal involvement, thus resulting in long-term health disparities [9,11-13].

The VA mental health residential rehabilitation treatment programs (MH RRTPs) [4] provide an average of 3 months of residential care to veterans experiencing homelessness, many of whom have high rates of COD and criminal legal involvement [14]. While MH RRTPs assist veterans with addressing homelessness, mental health, and substance use, they often do not provide specialized care for those with COD and criminal legal involvement. This is a concern as veterans with COD and criminal legal involvement enrolled in MH RRTPs are particularly vulnerable to poor treatment engagement and adherence [15,16], and have higher rates of being irregularly discharged (ie, leaving treatment prematurely) from MH RRTPs [17]. Retention in MH RRTPs and engagement in continuing care services reduce the likelihood of rehospitalization and other costly acute medical services [18,19]. However, criminal recidivism disrupts the continuity of care and increases costly acute care service use and, in turn, reentry into residential programs [20,21]. Other reports indicate that low engagement among veterans with criminal legal involvement is due to COD; competing behavioral health needs; and practical barriers such as transportation, homelessness, and misunderstanding of VA eligibility [6,16,22-30]. Because of the multifaceted behavioral health needs of this population coupled with poor treatment engagement, veterans with criminal legal involvement may benefit from more comprehensive, wraparound interventions (eg, a community-based intervention that provides a variety of services and treatment to support needs and care transitions) [31] to reduce service fragmentation, criminal recidivism, and improve behavioral health symptoms and functioning [32,33].

The Maintaining Independence and Sobriety through Systems Integration, Outreach and Networking (MISSION) intervention was developed to address the needs of veterans and civilians with a COD experiencing homelessness. MISSION is a multicomponent, evidence-based, wraparound treatment intervention that is delivered by a case manager and peer support specialist team and includes materials to support its implementation (eg, treatment manual and participant workbook). To ensure fidelity [34,35], MISSION integrates three evidence-based core components to address COD: (1) critical time intervention, a time-limited assertive community treatment that addresses situational and motivational barriers to engagement in care [36-38]; (2) dual recovery therapy (DRT), comprising psychoeducational sessions that provide integrated care for mental health and substance use (Ziedonis, DM, unpublished manual, May 2005) [39]; and (3) peer support, a peer curriculum of structured standardized sessions, supporting veterans use of community recovery activities (eg, attending mutual support groups) delivered by a person with lived experience, and facilitating homework assignments to reinforce knowledge discussed in groups [40-42]. While much evidence supports MISSION components individually, when combined, they complement each other in meeting the needs of individuals with CODs [31,43-48]. To address the unique needs of veterans and civilians with COD and criminal legal involvement, an adaptation of MISSION, the criminal justice (MISSION-CJ) version was developed (Pinals, DA, unpublished manual, April 2014 and Smelson, D, unpublished manual, June 2014). MISSION-CJ is guided by the risk-need-responsivity framework of offender rehabilitation, which posits that rehabilitation should target “criminogenic needs” (ie, modifiable factors that are robust predictors of recidivism) [49,50]. Thus, MISSION-CJ enhances the DRT curriculum with additional sessions to address antisocial attitudes and behaviors (Gaba, A, unpublished manual, January 2020).

### This Study

The MISSION-CJ intervention has been evaluated in other criminal legal settings (ie, reentry support and treatment court settings), and has evidenced reductions in substance use, mental health symptoms, and criminal recidivism [51-54]. However, MISSION-CJ has not been tested in VA MH RRTP settings. Considering the previous effectiveness of MISSION-CJ and the increasing need for comprehensive COD treatment for veterans involved in the criminal legal system, a critical next step is to understand how MISSION-CJ complements MH RRTP treatment to improve the behavioral health outcomes of veterans with criminal legal involvement and COD. Notably, MISSION-CJ provides an opportunity for a paradigm shift in VA care by offering a hybrid treatment and linkage intervention that simultaneously addresses recidivism and behavioral health outcomes and thus could improve care for veterans with criminal legal involvement. Therefore, the study described in this protocol paper will simultaneously test the effectiveness and implementation of MISSION-CJ in MH RRTPs for veterans with criminal legal involvement and COD.

## Methods

### Study Design

Using a hybrid type 1 effectiveness-implementation design [55], we will test the effectiveness of the MISSION-CJ intervention in a randomized controlled trial (RCT) across 2 large VA health care systems as well as conduct a qualitative evaluation of MISSION-CJ’s implementation. Hybrid type 1 designs are beneficial as they allow for simultaneously examining the effectiveness of an intervention (primary aim), mechanism analysis (secondary aim), as well as the intervention’s implementation potential (secondary aim) [54]. The *primary aim* of this study is to evaluate whether *MISSION-CJ in addition to the usual MH RRTP care* in comparison to *enhanced usual care (EUC)* lowers criminal recidivism and improves other health-related outcomes (ie, substance use, mental health, housing, and employment) and whether the effects of MISSION-CJ on recidivism and other health-related outcomes are mediated (secondary aim) by reductions in antisocial attitudes; reductions in affiliations with antisocial peers and increases in affiliations with prosocial peers; greater treatment engagement and service use (ie, MH RRTP completion, substance use or mental health continuing care, and mutual support group attendance); and increased community integration measured at 6 months and 15 months after baseline. In addition, the *secondary aim* of this study is to qualitatively evaluate MISSION-CJ using the RE-AIM (Reach, Effectiveness, Adoption, Implementation, and Maintenance) framework.

### Setting: VA MH RRTPs

Veterans will be recruited from VA MH RRTPs at 2 large VA health care systems in the United States. MH RRTPs provide treatment and rehabilitation services to veterans to address their complex behavioral health needs. Services are designed for improving functional status, sustaining treatment and rehabilitation gains, recovery, and community integration. At each VA site, we will recruit from a specific type of MH RRTP for veterans experiencing homelessness, which aims to improve their health and facilitate community reintegration by providing 24/7 care using a structured residential environment. MH RRTPs are comparable across VA sites in terms of program structure and services delivered; program duration (an average of 3 months); individual and group-based clinical approaches, such as cognitive behavioral programming; and staffing (psychiatrists, psychologists, social workers, nurses, addiction therapists, vocational therapists, and homelessness coordinators).

### RCT Participants

#### Overview

Veterans who (1) are entering an MH RRTP, at either of the 2 VA health care systems; (2) were arrested and charged or released from incarceration in the past 12 months; and (3) have a COD diagnosis in their medical record will be eligible for participation. Diagnoses will be confirmed during screening using the Structured Clinical Interview for the *Diagnostic and Statistical Manual of Mental Disorders, Fifth Edition* [56]. The only exclusion criterion is cognitive impairment, which will be assessed using the Montreal Cognitive Assessment’s section on Orientation. Veterans who are unable to correctly answer the date and location items will be deemed too cognitively impaired to understand the study’s procedures, participate meaningfully in the study, and respond to interviews, and therefore will be ineligible.

#### Sample Size Determination

To ensure sufficient variability in our outcomes and increase the power to test study hypotheses, power analyses were calculated to determine the sample size needed to obtain a medium-to-large effect size (based on the 3 open pilots with MISSION-CJ) with 80% power [57]. To account for having veterans at 2 sites, power analyses using G*Power were based on having 4 cells (2 conditions per site) [58]. A sample size of 180 (90 in each condition) will provide 91% power to detect a medium-large effect size at an α of .05 (2-tailed test). On the basis of an N of 180 and of .05, we would have 80% power to detect a Cohen *f*=0.25, a medium size effect [57]. Thus, after accounting for attenuation and 20% attrition [31,59,60], we will recruit 226 veterans (113 per condition). The sample will be stratified by site such that 113 veterans (with rounding) will be recruited separately from each VA site, with 57 veterans (with rounding) entering each condition at each site. The sites were selected because they have MH RRTPs that serve a high volume of veterans with criminal legal involvement and a COD; have criminal legal system outreach specialists, which will maximize recruitment by increasing the pool of veterans that could be referred to the MH RRTPs; and provide geographic variability, which will increase the generalizability of the findings.

#### Recruitment for RCT

MH RRTP staff will screen veterans during their admissions process to determine their eligibility. Potentially eligible veterans will be contacted by a research assistant who will explain the purpose of the study, procedures, and risks and benefits of participation. Veterans will be informed that they will be assigned to either an average of 2 hours of additional programming via a peer support group with community outreach care (EUC) or an intervention which provides an average of 2 hours of additional programming weekly, delivered by a MISSION-CJ treatment team. Veterans will be asked to complete an in-person baseline assessment and 2 follow-up assessments (in-person or via phone) 6 months and 15 months (ie, approximately 1 year post-MH RRTP discharge for those not irregularly discharged) later. Veterans will be compensated US $25 for completion of the baseline, US $50 for the 6-month assessments, and US $75 for the 15-month assessments.

### Randomization

After completion of the baseline assessment, 226 participants will be randomly assigned to either the EUC or MISSION-CJ condition. Randomization will be done using an SAS macro. Randomization will occur in blocks of 6 to prevent runs that might lead to unequal N’s between conditions if the full sample is not obtained. Following the baseline assessment, the research assistant will inform the data manager that a veteran has been enrolled. The data manager will add the veteran to the randomization spreadsheet, notify the veteran of their group assignment, and contact the EUC or MISSION-CJ providers to let them know a veteran has been randomized to their respective condition. The EUC or MISSION-CJ providers will then schedule the first session with the veteran. This process will ensure that the veteran’s condition remains blinded to the research assistant conducting assessments.

### Study Conditions

#### Overview

**Figure 1:**
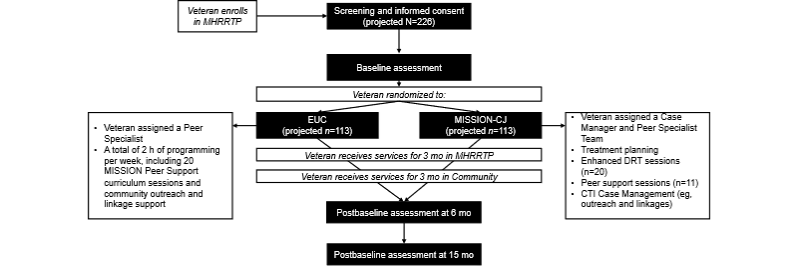
Randomized controlled trial (RCT) flow diagram. CTI: critical time intervention; DRT: dual recovery therapy; EUC: enhanced usual care; MHRRTP: mental health residential rehabilitation treatment program; MISSION-CJ: Maintaining Independence and Sobriety through Systems Integration, Outreach, and Networking Criminal Justice.

Veterans will be randomized to either MH RRTP care as usual (described in the previous section), with MISSION-CJ or EUC (refer to Table 1 for a comparison of the conditions and Figure 1 for a schematic flow diagram). EUC was chosen to provide an active control that was more ethically and clinically appropriate while still maintaining enough distinction between conditions to adequately test the added value of MISSION-CJ. Both conditions will be rolling entry.

**Table 1:** Comparison of the study conditions.

	MISSION-CJ^a^	Enhanced usual care
Treatment duration	6 months (begins within a week of the MH RRTP^b^ enrollment, duration of stay is 3 months, and continues for 3 months after the MH RRTP in the community) 2 hours of additional programming per week during months 1 to 3, weekly outreach sessions in month 4 to link veterans to prosocial community supports (eg, drug-free events and probation), biweekly sessions in month 5 (when the veteran is trying linkages), and 1 session in month 6 (MISSION-CJ discharge to transition care to linkages)	6 months (begins within a week of the MH RRTP enrollment, duration of stay is 3 months, and continues for 3 months after the MH RRTP in the community) 2 hours of additional programming per week for 6 months
Treatment services received by veterans	Risk-need-responsivity–based treatment planning tool Enhanced DRT^c^ (20 sessions) Peer support curriculum (11 sessions) Case management using the CTI^d^ step-down model to promote linkages to behavioral health and criminal legal services	MISSION Peer Support curriculum (24 sessions) Community outreach and linkage support
Treatment condition providers	Case manager and peer support specialist treatment team	Peer support specialist

^a^MISSION-CJ: Maintaining Independence and Sobriety through Systems Integration, Outreach, and Networking Criminal Justice version.

^b^MH RRTP: mental health residential rehabilitation treatment program.

^c^DRT: dual recovery therapy.

^d^CTI: critical time intervention.

#### Training for the MISSION-CJ and the EUC Conditions

At the outset of the study, the case manager and peer support specialist teams delivering MISSION-CJ will receive training on the curriculum, whereas the peer support specialist delivering EUC will receive training on the MISSION Peer Support curriculum as well as how to perform community outreach guidance. The training will be kept separate for each condition but will use the same format. The staff in both groups will first watch a 1.5-hour webinar on the MISSION-CJ or the EUC curriculum. This will be followed by a 1-day comprehensive training on the components of the MISSION-CJ or the EUC curriculum led by the MISSION developers. The MISSION-CJ training will review the components of the intervention, the risk-need-responsivity model, and the implementation materials. The EUC training will cover the MISSION Peer Support curriculum, community outreach, and the implementation materials. Furthermore, staff will have access to implementation materials (ie, the MISSION-CJ Treatment Manual or the MISSION Peer Support Enhanced Service Delivery Manual; Pinals, DA, unpublished manual, April 2014 and Smelson, DA, unpublished manual, August 2019).

### Data Collection

#### Data Collection for the RCT

Trained research assistants will administer a battery of assessments (Table 2) with veterans at 3 time points: baseline and at 6 months and 15 months after baseline. All data are entered and managed in REDCap (Research Electronic Data Capture; Vanderbilt University) [61,62]. Each assessment includes questions regarding substance use and mental health history, criminal history and recidivism risk, housing, employment, medication adherence, mutual help groups attendance, antisocial attitudes, affiliations with antisocial peers and prosocial peers, community involvement, and treatment services received.

**Table 2:** Measures for baseline and follow-up assessments for the primary aim.

Domain	Measure	Description	Time points
Substance use^a^	Timeline Follow-back	Retrospective, calendar-based measure; will provide information on quantity and frequency of substance use separately by drug type (ie, percent days of use and percent days abstinent) in the past 90 days [63]	Baseline, 6 months after baseline, and 15 months after baseline
Mental health^a^	Behavior and Symptom Identification Scale-24	Includes a total score and 6 subdomains (eg, depression and functioning, interpersonal relationships, and self-harm); will be used to measure change in mental health functioning [64]	Baseline, 6 months after baseline, and 15 months after baseline
Recidivism, recidivism risk and criminal history^a^	Level of Service Inventory–Revised	Validated and reliable scale consisting of 81 items that measure the dynamic risk factors that are summed to create an overall index of recidivism risk [65]	Baseline, 6 months after baseline, and 15 months after baseline
Housing status^b^	Residential Timeline Follow-back	Retrospective, calendar-based measure; will provide information on duration and frequency (eg, percent of days homeless) in the past 90 days [66-68]	Baseline, 6 months after baseline, and 15 months after baseline
Employment status^b^	Maudsley Addiction Profile (employment subscale)	Measures individuals’ employment status (eg, days worked, days absent, and days unemployed) using a 30-day recall period [69-74]	Baseline, 6 months after baseline, and 15 months after baseline
Medication adherence^b^	Medication Adherence Rating Scale	Measures veteran medication adherence behavior and how well the veteran adheres to medication [75-77]	Baseline, 6 months after baseline, and 15 months after baseline
Mutual support groups^c^	Alcoholics Anonymous Involvement	Adapted to measure attendance and involvement in mutual support groups for substance use (lifetime and past 12 months) [78]	Baseline, 6 months after baseline, and 15 months after baseline
Antisocial attitudes and peers^c^	Measures of Criminal Attitudes and Associates	Provides a valid and reliable assessment of an individual’s (1) antisocial attitudes (scale A) and (2) antisocial associates (scale B) [79-81]	Baseline, 6 months after baseline, and 15 months after baseline
Prosocial peers^c^	Community Assessment Inventory	Valid and reliable 10-item measure of support in 4 domains: household members, family outside home, friends, and community [75,82]	Baseline, 6 months after baseline, and 15 months after baseline
Community involvement^c^	Community Integration Measure	10-item valid and reliable measure of an individual’s integration into their home and community and is used in a broad range of disability research to assess client assimilation, support, productivity (vocational and leisure), and independent living [83-86]	Baseline, 6 months after baseline, and 15 months after baseline

^a^Primary outcomes.

^b^Secondary outcomes.

^c^Mediators.

#### Health Care Tracking

Health care tracking (mediator) will be conducted weekly and in 2 ways. First, self-reported treatment services received will be tracked via the MISSION Treatment Service Tracking Sheet, which includes detailed logs that quantify the amount and type of services received before and after the MH RRTP and is collected via REDCap. This includes all possible services offered within the MISSION-CJ and the EUC, as well as the community linkages. Regarding linkages, the measure quantifies the number of referrals, referral type, and client engagement. The MISSION-CJ and EUC providers will be prompted weekly to send their logs. The MISSION-CJ services received will be tracked via the MISSION-CJ Fidelity Scale. This scale consists of 27 items (scored yes or no) assessing adherence to the DRT and peer components (Pinals, DA, unpublished manual, April 2014). The fidelity for the MISSION Peer Support curriculum as part of the EUC will consist of a 13-item scale (scored yes or no), which also assesses model delivery adherence (Pinals, DA, unpublished manual, April 2014 and Smelson, DA, unpublished manual, August 2019). Both scales have demonstrated good interrater reliability (Pinals, DA, unpublished manual, April 2014 and Smelson, DA, unpublished manual, August 2019). For MISSION-CJ, we will also use the Critical Time Intervention Fidelity Scale, which includes 20 items rated on a 5-point scale, ranging from “not implemented” to “ideally implemented.” Item ratings can be combined to compute an overall fidelity score [87,88]. Second, additional data will be captured from the VA’s Corporate Data Warehouse (CDW) [89]. These data include MH RRTP completion status, which will be defined as a regular discharge from the program. Data on substance use and mental health continuing care use from the previous assessment will be defined as the number of sessions in each setting that the clients attend throughout the follow-up period, which will also be captured via the CDW. The CDW data includes both outpatient encounter records and information on inpatient and residential care services used. We will extract all health care encounters from the CDW that occur in a 12-month period before, and ending 15 months after, the study enrollment for all veterans.

#### Qualitative Evaluation

For the qualitative evaluation, we will interview 50% (12/24) of the veterans from each site who consented to participate in the study and were randomized to the MISSION-CJ group. Half of these veterans will be drawn from those considered high engagers (ie, completed a full dose, 6 months) of MISSION-CJ, and the other half will be drawn from those considered low engagers (ie, dropped out or completed half or less than the full dose, ≤3 months). The dose of the MISSION-CJ will be determined by the number of sessions attended by the veteran. We will also interview 50% (7/14) of the VA care providers (eg, nurses, psychiatrists, and social workers) from each site.

The RE-AIM Planning Tool will be used to conduct semistructured interviews with the MH RRTP veterans and care providers at each site, using 2 different sets of interview guides [90]. The RE-AIM framework highlights 5 domains to evaluate an intervention’s potential for implementation and widespread impact: *reach* (how to reach the target population with the intervention), *effectiveness* (how to know the intervention is effective), *adoption* (organizational support), *implementation* (fidelity of intervention’s delivery), and *maintenance* (sustainability) [91]. The RE-AIM Planning Tool asks questions to veterans and VA care providers within each domain regarding issues that should be considered when planning to implement an intervention (refer to Table 3 for sample items). Notably, we will supplement qualitative data with quantitative fidelity data to assess reach and adoption.

**Table 3:** Sample evaluation items by RE-AIM (reach, effectiveness, adoption, implementation, and maintenance) domain and participant.

RE-AIM domains	Veteran questions	VA care provider questions
Reach	How can we encourage other veterans to participate in MISSION-CJ^a^?	Which type of communication has tended to be most helpful for you to accomplish your MISSION-CJ related activities? To spread the word of the program?
Effectiveness	What changes did you notice in yourself while and/or after participating in MISSION-CJ?	To what extent was MISSION-CJ effective at actually meeting the needs of veterans with COD^b^ and criminal legal involvement?
Adoption	What was it like for you to work with the people on your MISSION-CJ team?	To what degree did people involved with MISSION-CJ buy-in to the intervention before it was implemented? Why?
Implementation	Did you receive materials during the MISSION-CJ program? What did you think of these materials? How often did you use them? What do you use the most and why?	How did it work to deliver MISSION-CJ along with your other responsibilities in your role here?
Maintenance	Would you recommend this program to a family member or a friend? Why?	What resources are available to sustain organizational support of MISSION-CJ, such as key decision maker commitments?

^a^MISSION-CJ: Maintaining Independence and Sobriety through Systems Integration, Outreach, and Networking Criminal Justice version.

^b^COD: co-occurring disorder.

### Ethics Approval

This study was reviewed and approved by the VA’s Central Institutional Review Board (#20-01) on February 25, 2021, and registered with ClinicalTrials.gov (NCT04523337). Consent will be obtained by a research assistant who has been trained in human participants protections and is knowledgeable about the study before participating in any study assessments or procedures. At the time of consent, the study will be explained in depth, and the prospective participant will be given the opportunity to have any questions answered to their satisfaction before being asked to provide informed consent. During this time, prospective participants will be informed that they will be assigned either to the EUC or to the MISSION-CJ (each of which will be described in detail). Prospective participants will be told that enrolling in the study is voluntary, and they may refuse to answer any questions and withdraw at any time; we will assure potential participants that their decision to withdraw it will not impact the standard of care they are receiving (eg, MH RRTP). For the qualitative evaluation, we will ask for verbal consent from both the veterans and the VA care providers (veterans will have provided written consent in the overall study’s consent form). In compliance with guidelines and oversight from the Data and Safety Monitoring Board, all collected data will be stored in a format that is identifiable only by an experimentally assigned ID number. The master list that contains identifiable information (eg, name and contact information) will be kept in restricted-access folders behind VA firewalls to which only the research team will have access. In addition the veterans will be compensated US $25 for the completion of the baseline, US $50 for the 6-month assessments, and US $75 for the 15-month assessments.

### Data Analysis

#### RCT Data Analysis

Before any outcome analysis, we will examine whether veterans assigned to the MISSION-CJ and the EUC conditions are comparable on sociodemographics (eg, age, race and ethnicity, marital status, education, and income) and baseline measures of the outcomes and mediators using independent group *t* tests and chi-square tests for continuous and categorical variables, respectively.

To test the effectiveness on the primary (ie, recidivism, risk for recidivism, substance use, and mental health) and secondary (ie, housing, employment status, and medication adherence) outcomes, we will use generalized linear mixed effects regression models to compare the EUC and MISSION-CJ conditions on outcomes. For each outcome, we will estimate a generalized linear mixed effects regression model that specifies the appropriate distribution of the outcome variable in the regression model (ie, normal, binomial, or Poisson) and uses the appropriate link and variance functions [92]. All models will include main effects for time (ie, baseline and 6- and 15-month follow-up), treatment (ie, MISSION-CJ vs EUC), and study sites, and will account for clustering of repeated measures over time within individuals using a random effect for veterans. We will assess the effectiveness of the intervention using a treatment x time interaction term, which will estimate the change in the outcome measure of interest across the 3 assessment points for the MISSION-CJ group as compared to the EUC group. All models will also be adjusted for all baseline characteristics to increase the precision of intervention effect estimates*.* Because the MISSION-CJ and EUC conditions will be standardized and monitored for fidelity, we do not expect variations in outcomes across sites. However, to address the possibility of contamination, we will ask the participants in the EUC group at the 6- and 15-month follow-up assessments whether they observe the MISSION-CJ information being shared in the MH RRTP. The records of the participants in the EUC group who say “yes” will be flagged, and the contamination-adjusted intent-to-treat analyses will be calculated (ie, the effect of treatment assignment on outcomes is adjusted by the percentage of participants assigned to the EUC condition who may have received the treatment) [93].

To minimize missing data, we will train research assistants on the importance of completing each item while respecting the veteran’s rights to refuse answers. On the basis of previous studies, we expect less than 5% missing data on each variable at each assessment, in addition to any attrition. We will obtain as high a follow-up rate as possible at each assessment. In contrast to the data on the MH RRTP completion and substance use or mental health continuing care use (which will be collected primarily via the VA administrative data at the 6- and 15-month follow-ups), and the data on recidivism (which will be collected as described previously), the data on recidivism risk and health-related outcomes at the follow-ups will be collected by our study team, and will therefore have more missing data due to expected attrition. The strengths of the generalized linear mixed effects regression model include its ability to use all available data, including data on respondents who have not provided data at each assessment. All the veterans who enter the study will be included in generalized linear mixed effects regression model analyses. However, we will also conduct analyses that include only those veterans who attended at least the first DRT and peer sessions. We will use a model-based multiple imputation procedure and impute missing values using least squares regression imputation [94]. We will select a set of measures associated with the variable at issue and use it in a series of iterated least squares regression models to generate a predicted value for the variable being imputed, and then we will substitute the missing value with the predicted value [95].

We will also examine whether and to what extent, reductions in antisocial attitudes, reductions in affiliations with antisocial peers, increases in affiliations with prosocial peers, MH RRTP completion, substance use or mental health continuing care engagement, mutual help group attendance, and community integration mediate the relationship between treatment group and recidivism, risk of recidivism and health-related outcomes at 6- and 15-month follow-ups [13,96]. To test each mediator, we will estimate a series of regression models that correspond to a hypothesized causal sequence among (1) MISSION-CJ, (2) one of our hypothesized mediators, and (3) better outcomes. In each model, we will control for relevant covariates (eg, baseline values for sociodemographic and the dependent variable). In the first model, we will regress the outcome variable of interest (eg, recidivism and the Level of Service Inventory–Revised [LSI-R] total scores) on the MISSION-CJ treatment condition. In the second model, we will regress the potential mediator of interest on the MISSION-CJ treatment condition. In the third model, we will regress the outcome of interest (eg, recidivism and the LSI-R total scores) on the potential mediator. If the coefficient for the MISSION-CJ treatment condition or a potential mediator is significant in all cases, we will proceed. In the final model, we will regress the outcome of interest (eg, recidivism and the LSI-R total scores) on the MISSION-CJ treatment condition and the potential mediator. If the coefficient for the potential mediator is significant and the coefficient for the MISSION-CJ dummy variable on the outcome is reduced, we will conclude that mediation is supported and will evaluate whether the indirect effect is significant.

#### Qualitative Evaluation

Deidentified audio files of the veteran and VA care provider interviews will be transcribed using VA Microsoft Teams and analyzed using NVivo (Lumivero) [97]. The research staff will review transcripts of these interviews, taking detailed notes using the RE-AIM Planning Tool structure to guide the analysis. Interviewers will record supplementary observations of initial impressions from the interview. Using an a priori approach informed by AIM combined with thematic analysis, research staff will jointly code several interviews to craft a codebook based on RE-AIM. Coding will involve close engagement with the data, multiple readings, and collaborative iterative defining of the codes [98-101]. We will then organize the codes using RE-AIM into broader themes. We will synthesize the codes into content categories and organize themes for final data interpretation. Drawing on our NVivo database and with team member feedback, we will create tables to summarize and categorize the barriers and facilitators of the MISSION-CJ implementation at the system, treatment care provider, and patient levels, including potential solutions and potential-to-leverage columns that contain evidence-based implementation tools to be considered for more widespread MISSION-CJ use. These analyses will reduce and display the data. With the tables, we will identify a small set of themes within each domain of the RE-AIM framework that signify triangulated findings (eg, factors that would facilitate and maintain use of MISSION-CJ that fit within the clinical and administrative infrastructure of a given facility). We aim to capture in-depth information on the patient, treatment provider, and contextual factors that facilitate or hinder the successful implementation of MISSION-CJ. We will use such factors to inform post hoc interpretation of the findings and guide the development of action plans to be tested in subsequent implementation projects. If hypotheses regarding the effectiveness of MISSION-CJ (vs EUC) are not supported, we will use this analytic approach to identify barriers in delivering MISSION-CJ during the RCT and to determine what modifications could be made to maximize effectiveness.

## Results

As of March 2025, 118 veterans (site 1: n=52, 44.1% and site 2: n=66, 55.9%) have been enrolled. In total, 58 veterans (site 1: n=27, 47% and site 2: n=31, 53%) have been randomized to the MISSION-CJ intervention and 60 veterans (site 1: n=25, 42% and site 2: n=35, 58%) have been randomized to the EUC group. The veterans are continuing to actively receive treatment and complete follow-up assessments. For the qualitative evaluation, we have completed 23 interviews with veterans. We have started transcribing these data and anticipate analyzing these data in the upcoming months. It is expected that by September 2025, all veterans will have ended the active treatment phase, and then for the following 9 months, we will continue conducting the remaining follow-up assessments. Parallel to this period, the remaining VA care provider interviews for the qualitative evaluation will be conducted, transcribed, and analyzed. We used the SPIRIT (Standard Protocol Items: Recommendations for Interventional Trials) checklist when writing this protocol (refer to Multimedia Appendix 1) [102]. The findings from this trial and qualitative evaluation should be available by 2026.

## Discussion

### Anticipated Findings

**Figure 2:**
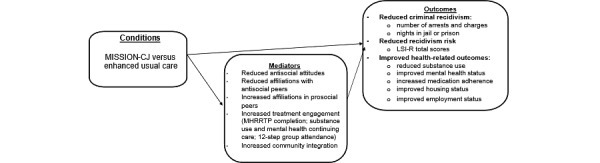
Logic model of study hypotheses. LSI-R: Level of Service Inventory-Revised; MHRRTP: mental health residential rehabilitation treatment program; MISSION-CJ: Maintaining Independence and Sobriety through Systems Integration, Outreach, and Networking Criminal Justice version.

This study is guided by the logic model depicted in Figure 2. Among justice-involved veterans with COD who are receiving care in an MH RRTP, we hypothesize that those randomized to the MISSION-CJ intervention (vs the EUC group) will have lower rates of criminal recidivism, lower recidivism risk, and improved health-related outcomes (ie, substance use, mental health, housing, and employment). Moreover, we hypothesize that the effects of MISSION-CJ on reduced recidivism and recidivism risk and better health-related outcomes will be mediated by reductions in antisocial attitudes; reductions in affiliations with antisocial peers and increases in affiliations with prosocial peers; greater treatment engagement (ie, MH RRTP completion, substance use or mental health continuing care use, and mutual support group attendance); and increased community integration. We anticipate that the information gained from the qualitative evaluation will provide guidance to our partners in terms of the broader adoption of MISSION-CJ in the MH RRTPs.

### Limitations

This study has several limitations. In particular, while the MH RRTPs were selected with input from key decision makers, this setting poses limitations. First, while veterans enroll in the MH RRTP treatment for 3 months, some veterans may be irregularly discharged due to violating program policies, or veterans may choose to leave the program prematurely. Though both conditions include treatment in the community, leaving the MH RRTP places veterans at risk for disengaging with treatment. Furthermore, leaving the MH RRTPs early disrupts rapport building with case Managers and peer support specialists, which can impact the quality of further treatment and engagement. Second, veterans enrolled in the MH RRTPs are likely to be receiving other individualized treatments, both within and outside of the VA, that may impact their outcomes. Finally, this study is limited to the experiences of veterans within the VA health care system, and therefore, findings may not be generalizable to veterans who are not receiving care in the VA; however, previous MISSION-CJ studies with veterans outside of the VA have found significant improvements [31,47,51,103].

### Conclusions

The MISSION-CJ intervention was developed to meet a critical gap by addressing the unique needs of individuals with criminal legal involvement. Having been found effective in other criminal legal contexts, coupled with the service gap in VA care and potential benefits to veterans, an essential next step is to test the effectiveness of MISSION-CJ in the VA MH RRTPs. Thus, this study will test a hybrid linkage and treatment approach currently unavailable in the VA, offering a paradigm shift, supporting care access, emphasizing peer support, and targeting recidivism as a primary outcome. If successful, MISSION-CJ could potentially supplement the MH RRTP care, offering a combined solution to improve behavioral health outcomes among a population of veterans with unique and often unmet needs.

## Appendix

Multimedia Appendix 1 SPIRIT checklist.

Multimedia Appendix 2 Peer-Review report from HSR-4 Mental and Behavioral Health, Health Services Research Parent IRG Office of Research & Development (VA Health Systems Research, US Department of Veterans Affairs, USA).

## Abbreviations

CDW: Corporate Data Warehouse
COD: co-occurring disorder
DRT: dual recovery therapy
EUC: enhanced usual care
LSI-R: Level of Service Inventory–Revised
MH RRTP: mental health residential rehabilitation treatment program
MISSION: Maintaining Independence and Sobriety through Systems Integration, Outreach and Networking
MISSION-CJ: Maintaining Independence and Sobriety Through Systems Integration, Outreach and Networking Criminal Justice version
RCT: randomized controlled trial
REDCap: Research Electronic Data Capture
RE-AIM: reach, effectiveness, adoption, implementation, and maintenance
SPIRIT: Standard Protocol Items: Recommendations for Interventional Trials
VA: Veterans Affairs

## Data Availability

Analyses for this study will be performed using the Veterans Health Administration (VHA) data. Deidentified data may be provided upon request pending ethics approval and in accordance with VHA guidelines and permissions.
