# Supplementary material for: Intervention for Justice-Involved Homeless Veterans With Co-Occurring Substance Use and Mental Health Disorders: Protocol for a Randomized Controlled Hybrid Effectiveness-Implementation Trial
Source: JMIR Res Protoc. 2025 Jul 18;14:e70750. doi: 10.2196/70750 (PMC12317293; doi:10.2196/70750)
Supplement: Multimedia Appendix 2 [file resprot_v14i1e70750_app2.pdf]

KEY SUMMARY POINTS:

- Well written; Highly responsive to critiques
- Proposal would build on three previous trials
- If the outcome is two or more variables (arrests, charges, nights in jail) there needs to be consideration of adjustment for multiple comparisons

DESCRIPTION (provided by applicant):

Background: Among the 146,000 Veterans released from correctional settings annually, approximately 60% have a co-occurring mental health and substance use disorder (COD). These individuals often access treatment inconsistently, resulting in increased antisocial activities and acceleration into unemployment and homelessness – strong predictors of reoffending. VHA Mental Health Residential Rehabilitation Treatment Programs (MH RRTPs) commonly serve justice-involved Veterans (JIVs) with an estimated 50% annually. JIVs receive assistance with their addiction and behavioral health needs, but MH RRTP programs do not directly address their antisocial behaviors and cognitions. Furthermore, MH RRTP discharge is a vulnerable transition and no national transitional approach facilitates Veteran engagement in prosocial community behaviors that maintain MH RRTP gains, and ultimately reducing revolving door service use. Maintaining Independence and Sobriety through Systems Integration, Outreach, and Networking-Criminal Justice version (MISSION-CJ) is a new case manager and peer delivered team-based treatment for JIVs with a COD. Three recent open pilots of MISSION-CJ showed reduced criminal recidivism, improved behavioral health outcomes and increased access and engagement in care. A randomized controlled trial (RCT) is a critical next step prior to dissemination. Significance/Impact: This application is responsive to the VHA MISSION Act, Veteran Care Priorities of Access to Care, Mental Health, and Health Equity. The project aims to (a) increase access and engagement in VHA and community-based care, (b) offer timely Veteran-centered care, and (c) improve the health and well- being of JIVs while reducing disparities. It also includes an implementation aim to support VHA learning. Innovation: While MISSION-CJ derives in part from an evidence-based treatment for homeless individuals (MISSION), it includes a new conceptual framework and numerous new and differentiating features for a CJ population including: (1) a treatment planning tool focused on criminogenic needs that monitors progress and tunes service delivery elements, (2) a prosocial treatment curriculum, and (3) tools/resources to address Veteran legal issues. With MISSION-CJ, this study attempts to change the practice paradigm and transform care for JIVs by moving beyond the current model of linking Veterans to VA care and tracking behavioral health outcomes, to a hybrid treatment/linkage approach that addresses criminogenic needs, supports engagement in VA and non-VA care, and targets recidivism as an outcome–the gold standard for CJ research. Specific Aims: Aim 1: An RCT will compare MISSION-CJ to EUC. We predict that those in MISSION-CJ will have (1a) lower criminal recidivism; (1b) lower overall risk for criminal recidivism; (1c) better health-related outcomes (substance use, mental health, housing, employment); and (1d) the effects of MISSION-CJ on 1a, 1b and 1c, will be mediated by (i) reductions antisocial attitudes, (ii) reductions in affiliations with antisocial peers and increases in affiliations with prosocial peers, (iii) greater treatment engagement (i.e., MH RRTP completion; substance use/mental health continuing care; 12-step group attendance), and (iv) increased community reintegration. Aim 2: A formative evaluation will identify barriers and facilitators to future implementation of MISSION-CJ in other MH RRTPs nationally. Methodology: This project will use a Hybrid Type 1 design. First, we will test the effectiveness of MISSION- CJ in a two-site RCT (Bedford and Palo Alto VAs) with 226 Veterans with a COD, admitted to an MH RRTP, and previously arrested and charged and/or released from incarceration in the past 12 months. Next, we will use the Reach, Effectiveness, Adoption, Implementation & Maintenance framework to conduct a formative evaluation with 7 providers and 12 Veterans at each site to inform future MISSION-CJ implementation. Next Steps/Implementation: Depending on the results of this study, we will work with our VACO operational partners and two HSR&D Centers of Innovation to conduct a large multisite implementation trial.

CRITIQUE 1

1. Significance.

The proposed project has considerable potential to ameliorate the risk of reoffending and moderate potential to improve other health outcomes in criminal justice-involved Veterans. The theoretical rationale for the MISSION-CJ intervention and results of previous uncontrolled studies are both strong. The results are likely to be helpful in improving the existing care offered to Veterans who seek care through residential treatment.

2. Innovation and Impact.

Innovation is moderate with respect to intervention components, including peer support and supported linkage to outpatient care. The content of MISSION-CJ and, most particularly, the focus on criminogenic thoughts and behavior are more innovative. Results of the study are likely to add significantly to the literature on treatment of criminal justice-involved patients.

3. Approach.

The effect of patient dropout from treatment is still a concern. The stated RRTP dropout rate of 20% is relatively low, but it would be reasonable to expect dropout would be higher for the target patients, as both substance use disorder and non-mandated criminal justice involvement carry increased risk. No information was offered about timing of dropout. Early dropout (i.e., before much of the treatment is received) would have a more significant effect than late dropout. A more nuanced evaluation of RRTP completion as an outcome (e.g., reasons for dropout) would help to inform whether and to what degree MISSION-CJ affected discharge. In the revised proposal, it is noted that EUC participants would also be offered continued services after dropout, which reduces this concern somewhat. Given the team's prior experience of poor attendance to the Moral Reconation Therapy sessions offered within RRTPs, it is surprising that attendance to MISSION-CJ sessions while in the residential setting is not a tracked outcome.

Contamination between conditions remains probable, either from peer support staff or between patients. It is not clear from the description whether peer support specialists will only participate in one treatment condition or offer care to both. Although fidelity to each condition will be measured, the measure description is silent on whether fidelity to EUC will include omission of the MISSION-CJ active elements, as is typically done. In addition, the investigators' response to the concern about cross-participant contamination is inadequate. Contamination during treatment that occurs during downtime or other programming (e.g., during other group therapy) is likely.

As noted below, there has been improvement in the description and choice of outcomes. In particular, the more comprehensive and objectively measured outcome of recidivism is a noted benefit. However, it is noted that the majority of other outcomes are self-report. Consideration should be given to enhancing these with objective (e.g., urinalysis tests) and observed (e.g., during peer support visits) measures where possible. A related problem is the measure of post-RRTP treatment "engagement," which is defined as visits attended. This could be substantially different from adherence, which is defined as visits attended as a proportion of visits scheduled. For example, patients who are highly symptomatic with substance use or other psychiatric symptoms may be recommended to intensive outpatient treatment (e.g., 3 days a week of service) while stable patients may be asked to attend only once a month. Attending three visits over three months has a different meaning in these two groups.

4. Feasibility (including Sampling, Project Timeline and Staffing).

The project seems to be feasible in timeline and staffing, and the communication plan is well thought-out. Although it appears that the sample in both Bedford and Palo Alto will be attainable, a contingency plan would be more reassuring.

5. Implementation.

The project includes a specific aim focused on implementation (Aim 2), which is appropriate and will be informative regardless of the effectiveness outcomes. The project is aligned with VHA goals for justice-involved Veterans, homeless Veterans, and improving treatment engagement. Strong enthusiasm for the project is offered in multiple letters of support, suggesting potential for sustainability.

6. Investigator Qualifications.

The research team is well-qualified and has a history of effective collaboration. There is strong expertise in implementation science.

7. Multiple PI Leadership Plan.

Drs. Smelson and Blonigen have complementary expertise and each is well-equipped to manage their respective sites.

8. Facilities and Resources.

No concerns.

9. Adequacy of Response to Previous Feedback Regarding the Proposed Study.

The proposal is very responsive to previous critiques. The investigators offer a more precise and thoughtful description of the data to be collected. These changes included more comprehensive measures of recidivism, specific healthcare outcomes assessed with dedicated measures, and additional/expanded measures of community engagement. There was also a more specific description of how this project differs from prior work with justice-involved Veterans, though focused primarily on the theoretical differences. The control intervention is improved, with enhancements to the peer support specialists' duties. Although there remains an attention imbalance with a case manager available to only one group, this design may be as close as is feasible. There is a better rationale for the use of RRTPs, although concerns remain with contamination between conditions (see above).

10. Protection of Human Subjects from Research Risk.

No concerns.

11. Inclusion of Women and Minorities in Research.

No concerns.

12. Budget and Period of Support.

No concerns.

13. Sharing Research Data (Data Management and Access Plan (DMAP)).

No concerns.

14. Overall Impression.

This revised proposal is responsive to prior critiques, especially with regard to equivalency of treatment arms and more comprehensive and specific outcome measurement. While some concerns remain that might adversely affect study integrity, these are largely minor.

15. Key Strengths.

1. Much-improved proposal with greater specificity of treatment components and outcomes.

2. Strong team that is likely to be able to conduct the quantitative and the qualitative components of the study.

3. Both positive and negative results will likely be informative and useful for VHA.

16. Key Weaknesses.

1. Potential dropout and its effect on study outcomes remains a relatively minor concern.

2. Contamination of active treatment elements remains a minor concern.

CRITIQUE 2

1. Significance.

Strengths: The proposal would build upon results from 3 open trials of MISSION-CJ, which have found positive results. The proposal provides a compelling argument for addressing recidivism within the Veteran population. The intervention incorporates the risk-need-recidivism model for guiding interventions to reduce recidivism risk. The proposal describes filling a need to address recidivism among Veterans transitioning from MH RRTP to the community. The proposal also attempts to modify the original MISSION protocol which showed efficacy in improving behavioral outcomes but not recidivism.

Weaknesses: No significant weaknesses.

2. Approach.

Strength: The Hybrid 1 design is appropriate to the purpose of the study. The inclusionary/exclusionary criteria are clear and reasonable. The power analysis appears to be accurate. Appropriate fidelity measures will be implemented to ensure adherence to the model. Proposed measures demonstrate acceptable psychometric properties. The plan for capturing recidivism data is adequate. The approach to addressing potential confounds in the models are appropriate.

Weaknesses: No significant weaknesses.

3. Implementation.

Strengths: The flexibility of the model in being wraparound or standalone is a strength for its potential implementation. The program is in alignment with national VA priorities and has endorsement from key partners, including the National Center for Homelessness Among Veterans. Other endorsements include Dr. Jennifer Burden, Deputy Director of MH RRTP and Dr. Jodie Trafton, Director of the Program Evaluation and Resource Center. The proposal details how it is unique versus existing studies in this area. The proposal notes input from operational partners that guided the dissemination in MH RRTP programs.

Weaknesses: No major weaknesses.

4. Innovation.

A strength is that the study does not exclude Veterans based upon mental health diagnosis, thus broadening the potential impact. However, the focus on Veterans in MH RTTP limits the findings to this population. The proposal is timely and addresses an area in need of new approaches. If successful, the program could offer a new model for reducing recidivism and result in a paradigm shift in servicing Veterans in MH RTTP.

5. Feasibility (including Sampling, Project Timeline and Staffing).

Power analysis and sample size are appropriate to the proposal aims. Recruitment goals are supported by prior studies and align with the required sample size. The proposal describes commitment from the proposed site with regard to personnel that will be implementing the program. The project timeline is reasonable to accomplish the aims.

6. Investigator Qualifications.

The team has a strong track record of research in this area. They are highly capable of completing this proposal. The biographical sketch for Susan Zickmund was largely blank.

7. Multiple PI Leadership Plan.

The leadership plan is appropriate and aligns to the expertise of the investigators.

8. Facilities and Resources.

The project has more than adequate resources to carry out the aims. The project includes two primary sites: Center for Healthcare Organization and Implementation Research (CHOIR) located at the Bedford VA Medical Center, the Center for Innovation to Implementation (Ci2i) at the VA Palo Alto Health Care System. The project would also utilize the Centralized Transcription Resources Program located at the VA Salt Lake City Health Care System, which is funded by HSR&D to provide transcription services for COINs.

9. Adequacy of Response to Previous Feedback Regarding the Proposed Study.

The proposal seemed thoroughly responsive to prior critiques.

The proposal was responsive to prior critiques by expanding potential mediators to including measures of community engagement and prosocial behaviors.

A concern was raised about the prior proposal including arrest records from only two states. The revised application plans to use VINELink, which includes current arrest and incarceration records. In addition, the investigators are pursuing an agreement to obtain arrest records from the Department of

Justice's National Crime Information Center and through Freedom of Information Act (FOIA) requests to obtain arrest records of participants in all states.

10. Protection of Human Subjects from Research Risk.

The benefits outweigh the potential risks. Potential risk is appropriately addressed.

11. Inclusion of Women and Minorities in Research.

The study will include women and minorities and describes estimates of enrollment among these groups based upon prior studies. The proposal describes reasonable steps to encourage Veteran retention in the program.

12. Budget and Period of Support.

The budget and period of support are appropriate.

13. Sharing Research Data (Data Management and Access Plan (DMAP)).

DMAP is appropriate.

14. Overall Impression.

This proposal addresses a need in the field by testing a novel intervention for reducing recidivism and improving psychosocial outcomes among Veterans in MH RTPP programs. The proposal builds upon promising open trial research of the intervention. The approach is well-designed. The research team has the expertise and capability to complete this project. The proposal has support from key champions and from the local sites. If successful, it could help to transform models of care for Veterans with co-occurring mental health issues who are transitioning to the community. There are some minor concerns about the approach.

15. Key Strengths.

1. Addresses need in the field.
2. Strong scientific rationale and support from prior research.
3. Strong research team.
4. Solid approach to the design and analysis.
5. Support from key leadership.

16. Key Weaknesses.

1. None.

CRITIQUE 3

1. Significance.

The current proposal targets the vulnerable population of justice involved Veterans with co-occurring disorders. If successful, the adoption of MISSON-CJ could help to reduce overall recidivism rates, risk and mental health outcomes of these individual who participate in MH RRTPs.

2. Innovation and Impact.

MISSION-CJ seems tailored to the MH RRTP inpatient setting and implementation speaks solely of scaling the intervention within MH RRTPs. Previous critiques have wondered why MH RRTPs will be the only source of participants and the response highlights the alignment of MISSION-CJ with the needs of those in the MH RRTP setting. While the rationale is reasonable, the fact that results from this RCT cannot be generalized beyond those entering MH RRTPs and that there would therefore be no plan to implement it beyond that setting dampens the impact somewhat.

3. Approach.

This is a hybrid type 1 study with an RCT of the impact of MISSION-CJ versus enhanced Usual Care on recidivism, risk of recidivism and mental health-related outcomes and a formative evaluation of MISSION-CJ using qualitative interviews of providers and Veterans to inform implementation and scalability. Recruitment of all participants will be from VA Mental Health Residential Rehabilitation Treatment Programs (MH RRTPs). The overall approach is reasonable, but there are a few questions and concerns.

Major:
Regarding the recidivism outcome, it is noted that this is operationalized as the number of arrests and charges and number of nights in jail or prison. It is unclear if arrests and charges will be lumped together or assessed separately, and there is no indication that any adjustment for multiple comparisons will be made. Additionally, with appropriate adjustment, there is likely an impact on power.

Minor:
While the overall analytic plan is appropriate, the treatment by time interaction term is not applicable to the recidivism outcomes as they will be a single value assessed across the timeframe of interest.

It is unclear why the CDW will be used to determine MH RRTP completion as the participants will transition out of the RRTP during the study. Wouldn’t there simply be record of the completion based on the study itself?

4. Feasibility (including Sampling, Project Timeline and Staffing).

The project appears to be feasible given the timeline, staffing and rate of Veterans entering the MH RRTPs.

5. Implementation.

Planning of the current proposal included key stakeholders and operational partners which increases the probability of implementation if the study is successful. Investigators also note that MH RRTPs have staffing that is compatible with MISSION-CJ which will reduce additional necessary resources for implementation.

6. Investigator Qualifications.

The investigative team is well qualified to carry out the study.

7. Multiple PI Leadership Plan.

The PIs have complementary expertise and the plan is well laid out.

8. Facilities and Resources.

The facilities/resources are more than adequate.

9. Adequacy of Response to Previous Feedback Regarding the Proposed Study.

The investigators were responsive to previous critiques. The response regarding the MH RRTP setting is reasonable, the ASI was dropped, additional validated measures were added to replace the ASI as well as The Community Assessment Inventory, and Community Integration Measure. There is now a plan to use VINELink to capture arrests made outside of CA and MA, although it is unclear if individuals must be convicted (and not just arrested) to show up in this database (proposal states 'only those currently incarcerated'). It is also unclear if once all data from VINELink is collected, the requests will be made to all states that are included in that VINELink data capture. The adjustments to the control group are also well reasoned.

10. Protection of Human Subjects from Research Risk.

No concerns.

11. Inclusion of Women and Minorities in Research.

The study will include women and minorities – no concerns.

12. Budget and Period of Support.

No concerns.

13. Sharing Research Data (Data Management and Access Plan (DMAP)).

The DMAP is reasonable.

14. Overall Impression.

The overall study design is appropriate, and the study team was responsive to prior critiques. There are a few remaining concerns.

15. Key Strengths.

1. Strong leadership support.

2. Responsive to prior critiques, with multiple improvements and well-reasoned responses.

3. Appropriate study design.

16. Key Weaknesses.

1. No adjustments planned for the multiple comparisons of the primary recidivism outcomes.

2. More clarity needed regarding the added value of VINELink.

MEETING ROSTER

HSR-4 Mental and Behavioral Health
Health Services Research Parent IRG
Office of Research & Development
HSR4
08/22/2019 - 08/23/2019

CHAIRPERSON(S)

COHEN, AMY N., PHD
CO-DIRECTOR, HEALTH SERVICES, VA DESERT PACIFIC MIRECC
GREATER LOS ANGELES VA HEALTHCARE CENTER
ASSOCIATE RESEARCH PSYCHOLOGIST
UCLA CENTER FOR HEALTH SERVICES AND SOCIETY
LOS ANGELES, CA 90073

MEMBERS

BELSHER, BRADLEY E, PHD *
CHIEF OF RESEARCH TRANSLATION AND INTEGRATION
DEPT. OF PSYCHIATRY, USUHS
CHIEF OF RESEACH AND INTEGRATION
DEPLOYMENT HEALTH CLINICAL CENTER
DEFENSE CENTER OF EXCELLENCE FOR PH AND TBI
SILVER SPRING, MD 20910

BROWN, GREGORY K, BS, MS, PHD *
RESEARCH PSYCHOLOGIST
PHILADELPHIA VA MEDICAL CENTER
RESEARCH ASSOCIATE PROFESSOR, DEPT. OF PSYCHIATRY
UNIVERSITY OF PENNSYLVANIA
PHILADELPHIA, PA 19104

BRYAN, CRAIG J., BS, MS, PSYD *
ASSOCIATE PROFESSOR
NATIONAL CENTER FOR VETERANS STUDIES
ASSOCIATE PROFESSOR, DEPARTMENT OF PSYCHOLOGY
UNIVERSITY OF UTAH
SALT LAKE CITY, UT 84112

CEREL, JULIE A, BA, MA, PHD *
PROFESSOR
COLLEGE OF SOCIAL WORK
UNIVERSITY OF KENTUCKY
LEXINGTON, KY 40506

COMTOIS, KATHERINE ANNE JR, MPH, PHD *
PROFESSOR
DEPARTMENT OF PSYCHIATRY & BEHAVIORAL SCIENCES
ADJUNCT PROFESSOR
DEPARTMENT OF PSYCHOLOGY
UNIVERSITY OF WASHINGTON
SEATTLE, WA 98195

DAVIS, LORI L., MD *
ASSOCIATE CHIEF OF STAFF
TUSCALOOSA VA MEDICAL CENTER
CLINICAL PROFESSOR OF PSYCHIATRY
SCHOOL OF MEDICINE
UNIVERSITY OF ALABAMA
TUSCALOOSA, AL 35404

FINLEY, ERIN P, BA, PHD *
RESEARCH HEALTH SCIENTIST
AUDIE L. MURPHY MEMORIAL VETERANS HOSPITAL (ALMD)
ASSOCIATE PROFESSOR
DEPARTMENTS OF MEDICINE AND PSYCHIATRY
UNIVERSITY OF TEXAS HEALTH SCIENCE CENTER
SAN ANTONIO, TX 78229

FORSTER, JERI E, PHD
DATA & STATISTICAL CORE DIRECTOR
ROCKY MOUNTAIN MIRECC
EASTERN COLORADO VA MEDICAL CENTER
ASSISTANT PROFESSOR, DEPT. OF PHYSICAL MED & REHABILITATION,UNIV OF COLORADO SCHOOL OF MEDICINE
DENVER, CO 80220

FRENCH, DUSTIN D., PHD
SENIOR RESEARCH SCIENTIST
EDWARD HINES, JR. VA HOSPITAL
ASSOCIATE PROFESSOR
DEPT. OF OPTHALMOLOGY
NORTHWESTERN UNIV. FEINBERG SCHOOL OF MEDICINE
CHICAGO, IL 60141

GRADUS, JAIMIE L., BA, MPH, SCD *
EPIDEMIOLOGIST
VA BOSTON HEALTHCARE SYSTEM
DEPARTMENT OF EPIDEMIOLOGY
BOSTON UNIVERSITY SCHOOL OF PUBLIC HEALTH
BOSTON, MA 02118

HAAS, GRETCHEN L, PHD
ASSOCIATE DIRECTOR, VISN 4 MIRECC
VA PITTSBURGH HEALTHCARE SYSTEM
ASSOCIATE PROFESSOR OF PSYCHIATRY AND PSYCHOLOGY
WESTERN PSYCHIATRIC INSTITUTE AND CLINIC
UNIVERSITY OF PITTSBURGH MEDICAL CENTER
PITTSBURGH, PA 15213

HAMNER, MARK B., MD *
DIRECTOR, PSYCHOPHARMACOLOGY RESEARCH AND MEDICAL DIRECTOR, PTSD CLINICAL TEAM (PCT)
RALPH H. JOHNSON VA MEDICAL CENTER
PROFESSOR
DEPARTMENT OF PSYCHIATRY AND BEHAVIORAL SCIENCES
MEDICAL UNIVERSITY OF SOUTH CAROLINA
CHARLESTON, SC 29425

MCINTOSH, SCOTT, BS, MA, PHD *
ASSOCIATE PROFESSOR
DEPARTMENT OF PUBLIC HEALTH SCIENCES
UNIVERSITY OF ROCHESTER MEDICAL CENTER
ROCHESTER, NY 14642

PEIRCE, JESSICA M, BA, MS, PHD *
ASSOCIATE PROFESSOR
DEPARTMENT OF PSYCHIATRY AND BEHAVIORAL SCIENCES
JOHNS HOPKINS UNIVERSITY SCHOOL OF MEDICINE
BALTIMORE, MD 21224

PFEIFFER, PAUL NELSON, MD *
RESEARCH INVESTIGATOR
ANN ARBOR HEALTHCARE SYSTEM
ASSOCIATE PROFESSOR
UNIVERSITY OF MICHIGAN
ANN ARBOR, MI 48109

POLUSNY, MELISSA A., BA, LP, PHD *
STAFF PSYCHOLOGIST/CLINICAL INVESTIGATOR
CENTER FOR CHRONIC DISEASE OUTCOMES RESEARCH
MINNEAPOLIS VA HEALTH CARE SYSTEM
ASSOCIATE PROFESSOR, DEPARTMENT OF PSYCHIATRY
UNIVERSITY OF MINNESOTA MEDICAL SCHOOL
MINNEAPOLIS, MN 55417

RAMCHAND, RAJEEV N, BA, PHD *
ADVISOR FOR MENTAL HEALTH SERVICES
COHEN VETERANS NETWORK
SILVER SPRING, MD 20910

SCHUMM, JEREMIAH, BS, MA, PHD
ASSOCIATE PROFESSOR
SCHOOL OF PROFESSIONAL PSYCHOLOGY
WRIGHT STATE UNIVERSITY
DAYTON, OH 45435-0001

WILLIAMS, EMILY CATERINA, BA, MPH, PHD *
ASSOCIATE PROFESSOR
VA PUGET SOUND HEALTH CARE SYSTEM
ASSOCIATE PROFESSOR, DEPARTMENT OF HEALTH SERVICES
DIRECTOR OF DOCTORAL PROGRAM IN HEALTH SERVICES
UNIVERSITY OF WASHINGTON SCHOOL OF PUBLIC HEALTH
SEATTLE, WA 98101

WRAY, LAURA ODELL, PHD *
EXECUTIVE DIRECTOR, CIH
VA WESTERN NY HEALTHCARE SYSTEM
VA CENTER FOR INTEGRATED HEALTHCARE
ASSOCIATE PROFESSOR, DIVISION OF GERIATRICS
UNIVERSITY AT BUFFALO
BUFFALO, NY 14215-1129

SCIENTIFIC REVIEW OFFICER

O'BRIEN, ROBERT W., PHD
DEPARTMENT OF VETERANS AFFAIRS
VETERANS HEALTH ADMINISTRATION
OFFICE OF RESEARCH AND DEVELOPMENT
HEALTH SERVICES RESEARCH & DEVELOPMENT
WASHINGTON , DC 20420

* Temporary Member. For grant applications, temporary members may participate in the entire meeting or may review only selected applications as needed.

Consultants are required to absent themselves from the room during the review of any application if their presence would constitute or appear to constitute a conflict of interest.
